# Healthy eating and active living for diabetes in primary care networks (HEALD-PCN): rationale, design, and evaluation of a pragmatic controlled trial for adults with type 2 diabetes

**DOI:** 10.1186/1471-2458-12-455

**Published:** 2012-06-19

**Authors:** Steven T Johnson, Clark Mundt, Allison Soprovich, Lisa Wozniak, Ronald C Plotnikoff, Jeffrey A Johnson

**Affiliations:** 1 Centre for Nursing and Health Studies, Athabasca University, Athabasca, AB, Canada; 2 School of Public Health, University of Alberta, Edmonton, AB, Canada; 3 Alliance for Canadian Health Outcomes Research in Diabetes, University of Alberta, Edmonton, AB, Canada; 4 School of Education, University of Newcastle, Callaghan, NSW, Australia

**Keywords:** Type 2 diabetes, Primary care, Physical activity, Diet, Exercise specialist, Health service research

## Abstract

**Background:**

While strong and consistent evidence supports the role of lifestyle modification in the prevention and management of type 2 diabetes (T2DM), the best strategies for program implementation to support lifestyle modification within primary care remain to be determined. The objective of the study is to evaluate the implementation of an evidence-based self- management program for patients with T2DM within a newly established primary care network (PCN) environment.

**Method:**

Using a non-randomized design, participants (total N = 110 per group) will be consecutively allocated in bi-monthly blocks to either a 6-month self-management program lead by an Exercise Specialist or to usual care. Our primary outcome is self-reported physical activity and pedometer steps.

**Discussion:**

The present study will assess whether a diabetes self-management program lead by an Exercise Specialist provided within a newly emerging model of primary care and linked to available community-based resources, can lead to positive changes in self-management behaviours for adults with T2DM. Ultimately, our work will serve as a platform upon which an emerging model of primary care can incorporate effective and efficient chronic disease management practices that are sustainable through partnerships with local community partners.

**Clinical Trials Registration:**

ClinicalTrials.gov identifier: NCT00991380

## Background

Globally, 346 million people have diabetes and the prevalence of type 2 diabetes mellitus (T2DM) is expected to increase to an estimated 440 million in the next 20 years [1,2]. Consequently, treating and managing this population will be a substantial burden to health care systems [3]. There is, however, sufficient evidence to suggest that drug treatment, physical activity (PA), medical nutrition therapy, and body weight management can mitigate longer-term complications of diabetes [2] and thus potentially lessen the impending public health burden. Healthy eating and active living are two main ingredients of T2DM self-management. Attention to both nutrition and physical activity (exercise) has repeatedly and consistently been shown to be associated with improvements in metabolic risk [4-7]. As such, guidelines for healthy eating and active living have been developed for use in clinical practice [2,8]. Despite the availability of evidence-based guidelines, the majority of adults with T2DM follow unhealthy dietary patterns and are insufficiently active [9,10] suggesting current frontline approaches to promoting and supporting healthy self-management may not be effective or efficient. Several community-based or ‘real world’ interventions have demonstrated that self-management programs can be effective in improving behavioral and clinical outcomes [11-16]. Although these real world examples have shown success, questions still remain around the longer-term impacts of these interventions at the patient level and at the system level (i.e., primary care setting). More specifically, if patient exposure needs to be increased (i.e., program intensity) to sustain shifts towards positive self-management behaviours seen in what might be considered the early stages of adoption (e.g., 3 months), what are the longer-term costs within the context of sustainability for the patient and, at the same time, within the context of program delivery?

Acknowledging that gaps still exist in terms of our understanding of real world exemplars of diabetes self-management delivered in primary care, we designed the Healthy Eating and Active Living for Diabetes in Primary Care Networks (HEALD-PCN) study to explore questions around the effectiveness and efficiency of delivering an evidence-based self-management program linked with community resources within in a newly emerging model of primary care. We intend to develop a comprehensive understanding of the system requirements for implementation, which will in turn provide supportive evidence to inform policy makers with respect to resource allocation and the potential for program sustainability.

The main objective of HEALD-PCN is to evaluate a novel implementation of an evidence- based self-management program for patients identified as having T2DM within an established Primary Care Network environment in Alberta, Canada. The primary study hypothesis is that those allocated to HEALD-PCN program will self-report higher levels of moderate and vigorous physical activity and objectively monitored daily pedometer steps. Secondary objectives are to complete a comprehensive evaluation to understand why the program did or did not have an impact, identify critical factors to successful implementation and to develop recommendations to mitigate barriers to successful implementation if the intervention proves effective.

## Methods/design

### Ethical approval

All study procedures received approval from the University of Alberta Health Research Ethics Board prior to study commencement. All participants provided written informed consent.

### Setting & population

This research will be carried out in four non-urban Primary Care Networks (PCNs) in Alberta, Canada. All PCNs are stand-alone physician-led corporations established to support family physicians through allied professionals, including: Chronic Disease Management Nurses, Registered Dietitian, Pharmacists, Health Promotion and Prevention Coordinators and Exercise Specialists. According to the Alberta Diabetes Surveillance System, the prevalence of diabetes in the province of Alberta was 5.5% in 2009 with approximately 20% over the age of 65 [17]. In the four participating PCNs, approximately 9,000 adult patients with diabetes are under the care of approximately 140 General Practitioners.

All PCNs in the current study provide some form of basic diabetes group education either through a group-based workshop or one-on-one counseling with a Registered Dietitian and/or Exercise Specialist by physician referral. It is recognized, however that these approaches to support lifestyle self-management are most likely insufficient to achieve and maintain lifestyle changes in the longer term. Thus, the HEALD program is intended to be an enhanced follow-up behavioral support program, prescribed for patients who have already received some form of basic diabetes self-management education. The HEALD-PCN evaluation will be over and above the baseline self-management education through a pragmatic controlled trial [18].

### Study subjects & recruitment

To satisfy the main objectives and achieve the study aims, we propose to implement and evaluate the HEALD program among recently diagnosed patients who are cared for within the PCN environment. One component of the PCNs management structure was the development of a chronic disease patient registry. More generally, a unique PCN patient registry will include information about the patient’s medical management and allow physicians and PCN staff to track patient progress. In keeping with the primary care network patient chronic disease management strategy (i.e., tracking), potentially eligible patients will be identified and recruited through a patient registry.

Following identification of potential study participants through each participating PCN, an endorsement letter from each PCN will be mailed to the potential participant by staff located at each PCN. Staff with this research responsibility within each of the PCN environments is intended to simulate real world chronic disease management practices (i.e., recruitment to available PCN programs). The endorsement letter was selected as a recruitment option after consultation with the PCN chronic disease management teams as a means to create a credible link between the PCN and their clients. Those interested in the study will be asked to contact the PCN by telephone where they will be prescreened to further determine eligibility. For those who have not responded by telephone within a specified time, a PCN staff member will perform a follow up phone call to determine interest and eligibility. Once an individual is deemed eligible, they will be sent a study package, which will include: a study information sheet endorsed by the local PCN physician lead for chronic disease management, a consent form and a pedometer with instructions. A baseline appointment will then be made with a trained Exercise Specialist to collect all relevant baseline clinical/laboratory information at each centralized PCN location using point-of-care instruments.

Inclusion criteria

· On primary care patient registry (i.e., under PCN physician care);

· 18-75 years of age or who are capable of sustained walking for 10 minutes;

· Diagnosed with type 2 diabetes (self-reported and confirmed through PCN patient registry);

· Received basic diabetes education since being diagnosed;

· Proficient English language skills.

Exclusion criteria

· Unable to provide informed consent or unwilling to participate in the study;

· Unable to read, understand, and converse in English;

· Significant cardiovascular contraindications (self-reported history when prompted);

· Significant current depression (self-reported) Patient Health Questionnaire-9;

· Currently enrolled in any other study.

#### Patient allocation

**Figure 1:**
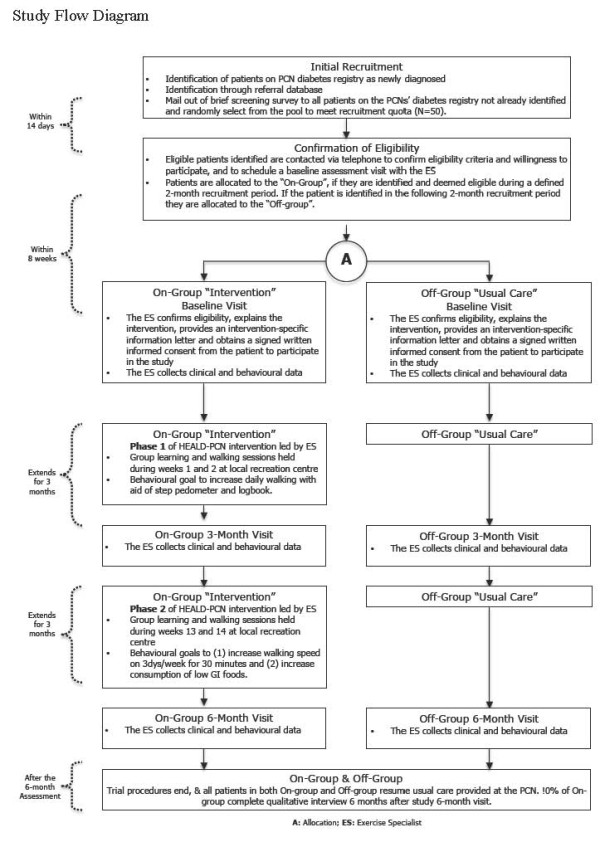
Study flow diagram.

Patients will be allocated to a control group (i.e., usual care) or the intervention group using an “On-Off” group assignment method (Figure 1) This method has been previously used in quality improvement studies [19-21], and meets study design quality criteria to permit inclusion in the Cochrane Collaborations’ Effective Practice and Organization of Care (EPOC) systematic reviews [22]. Conceptually, eligible PCN patients will enter a “recruitment pool” then allocated to the intervention or control group in sequential clusters. This recruitment cluster will be filled through a number of strategies, with an aim of 50 participants at a time. The first strategy will be through active identification of newly diagnosed patients on each of the PCN’s diabetes patient registry. It is recognized that not all newly diagnosed patients will be immediately identified through this registry and therefore as a second strategy, we will recruit newly diagnosed patients through other PCN referral registries (i.e., patients attending basic diabetes education classes). Newly diagnosed patients will be given priority entry into the intervention since they may be more likely to adopt new behaviours versus those having diabetes for a longer period of time.

Cut-point dates will be set to stop filling the recruitment pool over a two-month time frame (i.e., bi-monthly block). If the recruitment cluster fails to fill with newly diagnosed patients (i.e., less than 50 eligible), a third strategy for recruitment will be implemented which will have the remaining program invitation spots randomly selected from a second pool of patients who were contacted through an earlier pre-screen mail out to all diabetic patients on the PCN patient registry.

#### HEALD-PCN intervention

The HEALD-PCN intervention is based on a previously pilot-tested self-management program in this patient population [23]. It consists of two phases, lasting a total of 24 weeks, with emphasis on physical activity (walking) and nutritional elements. During weeks 1 to 12 (phase 1), participants allocated to the intervention group will be placed in a self-monitored, pedometer-based walking program targeting total daily steps. With initial guidance from an Exercise Specialist, each participant will set his or her own daily step goals. A baseline average will be calculated from three consecutive days including one weekend day [23-25] to help participants set individualized goals. The program goal for all participants over the first 12 weeks (phase 1) will be to increase their number of steps/day.

As part of phase 1, during weeks 1 and 2, participants will attend a 30-minute, group-based meeting, including a supervised walking session, facilitated by the Exercise Specialist, and located at a community recreation facility. A pedometer, a resource manual and step logbook will be provided at the first meeting to facilitate goal setting and to record the total number of steps/day. Access to the community recreation facility is based on partnership arrangements between the research team, the PCN and the local recreation facility management. Financial arrangements for these agreements are provided by the research funding. After week 12 (phase 2), participants will be asked to continue to walk the same number of steps/day that they walked during weeks 10-12 of phase 1. As part of phase 2, during weeks 13 and 14 participants will attend two more group-based meetings at the same community recreation facility, where they will be taught by the PCN Exercise Specialist how to increase their walking speed by 10% during a 30 minute walk. The participants will be asked to incorporate this faster walking pace for 30 minutes/day on 3 days/week on their own, until the end of the study period. For example, if a participants’ self-selected walking pace was 90 steps/minute, they will be encouraged to increase his or hers’ pace to approximately 100 steps/minute. Participants will be asked to perform their faster walking in bouts lasting no less than 10 minutes, and will be given a second pedometer and a stopwatch to help them measure and monitor their brisk steps and time. A second resource manual will be given to accompany this part of the program, which will include a set of small portable cards to record the number of brisk steps immediately after they are performed.

The nutritional element of the HEALD-PCN program will focus on the concepts of the Glycemic Index (GI) in accordance with the Canadian Diabetes Association Clinical Practice Guidelines [2]. The goal will be to increase daily consumption of low-GI foods. Participants will be encouraged to exchange high-GI foods with low-GI foods (i.e. replace white or whole wheat bread with pumpernickel) on at least 3 days/week and to make at least 2 exchanges over the course of those days. Glycemic index goals will be based on the Good-Better-Best principle proposed by Brand-Miller and Foster-Powell [26] which suggests a “this-for-that” exchange system whereby low-GI foods are euphemistically termed leakers (i.e., the “this”) should replace high glycemic index foods termed gushers (i.e., the “that”) in the diet. Achieving high task self- efficacy for identifying low and high glycemic index foods is considered an essential component of this study. We have previously shown this approach increases general GI knowledge [27].

#### Program delivery training for exercise specialists

One Exercise Specialist (i.e., certified by the Canadian Society of Exercise Physiology) will administer the intervention at each PCN. A full day workshop for all specialist detailing the delivery of the HEALD intervention will be organized and led by Dr. S. Johnson (developer of the HEALD intervention). Dr. S. Johnson will also meet with each Exercise Specialist at each PCN to review the HEALD materials prior to the delivery of the first HEALD group session. Study manuals that cover all aspects of the HEALD intervention will be provided at the training workshop.

#### Usual care

Participants in the usual care or “Off” group will receive usual or standard care for diabetes from their family physicians and/or chronic disease management team based on clinical practice guidelines. Participants in this group will be followed up for the same time (i.e., 6 months) and will undergo all the assessments and measurements as participants in the intervention group.

#### Study measures

We will use a combination of metrics to determine the effectiveness of the HEALD-PCN. As in our HEALD pilot study [23], the primary outcome is physical activity determined by self- report and pedometers. Secondary outcomes include nutrition behaviours (healthy eating and glycemic index), anthropometric assessments, and biomarkers. All primary and secondary outcomes will be collected at baseline, 3 and 6 months, through surveys and point of care instrument testing. Demographic, health/medical and other related characteristics will also be assessed at baseline, 3 and 6 months. At study completion, we will assess self-reported health status and satisfaction with the intervention materials and pedometers. We will also collect information on the cost of this enhanced lifestyle program in the PCN environment and subsequent participant health care utilization, through linkages with Alberta administrative data. To do so, participants enrolled in the study will be asked for permission to access their medical records by signing a consent form and providing their personal health number, thus allowing linkage to Alberta Health and Wellness physician billing, hospital billing, and emergency room billing data. This linkage will allow health care utilization and health care costs to be included in the evaluation.

##### Physical activity behaviours

Our primary outcome is self-reported physical activity, assessed with the Godin Leisure Time Exercise Questionnaire (GLTEQ) [28], modified to calculate MET minutes values [29] for total moderate and vigorous physical activity. The GLTEQ asks participants to report the average number of times per week, in the past month, they engaged in vigorous (rapid heartbeats, sweating), moderate (not exhausting, light perspiration) and mild (minimal effort and no perspiration) intensity PA, for a minimum of 10 minutes per session. Participant responses for the moderate and vigorous activity categories will be added together to calculate moderate and vigorous physical activity (MVPA) minutes per week and will serve as our primary self-report outcome measure.

For walking specific behaviors, pedometers (Yamax SW-200) and step logs will also serve as an objective measure of our primary outcome. Participants in both the intervention and control groups will be provided with a pedometer, and instructed to wear it and record total daily step-counts in a log for 3 consecutive days including one weekend day. The step log also allows for the collection of brisk steps for the intervention group. Self-reported walking will also be assessed using questions form the International Physical Activity Questionnaire (IPAQ) long version [30]. Participants will be asked, “During the last 7 days on how many days did you walk for at least 10 minutes at a time to go from place to place” or “in your leisure time”. Participants will then be asked, “How much time did you usually spend on ONE of those days walking from place to place” or “walking in your leisure time”. Responses will be recorded in days, hours, and/or minutes per day. A walking score will be calculated by multiplying the number of walking days by the walking minutes then by 3.3 (the MET value suggested for walking) to obtain walking scores in MET-minutes. Stages of physical activity behaviour change measures (≥ moderate levels) initially developed by Reed and colleagues [31] will serve as a secondary behavioral outcome (to assess stage transition).

##### Clinical measures

Using previously validated point of care testing devices, participants will have fasting capillary blood samples collected to assess hemoglobin A1c (DCA Vantage), lipid profile, glucose (Cholestech LDX), resting heart rate and blood pressure (BPTru). These clinical measures will be taken at baseline, 3 and 6 months. Anthropometric measurements including weight, height, and waist and hip circumference will be completed and body mass index (BMI) calculated.

##### Other measures

For nutrition behaviours, a validated food frequency questionnaire [32] will be used to estimate saturated fat, trans fat, total sugars, "added sugars" (in sweetened cereals, soft drinks, and sweets), fruit and fruit juice, vegetable intake, glycemic load and glycemic index. Validated published measures of health related quality of life [33-35], depressive symptoms using the Patient Health Questionnaire-9 [36-38], problem areas in diabetes [39], medication use, foot care, smoking behaviour, history of chronic diseases and risk factors [40]. Social cognitive variables related to physical activity behaviors from protection motivation theory, theory of planned behaviour, social cognitive theory, and stages of change will also be examined at baseline, 3 and 6 months using validated measures [41].

##### Implementation evaluation

By applying the RE-AIM Framework, our academic group and PCN partners are highly engaged in facilitating an in-depth understanding of the process variables necessary to inform the successful future adoption and sustainability of this intervention in similar primary care settings. The RE-AIM framework provides a systematic means to evaluate the overall population-based impact of an intervention and it’s potential for translation through five components that describe: Reach, Effectiveness, Adoption, Implementation, and Maintenance [42].

Unlike studies focused only on efficacy, the RE-AIM framework emphasizes both internal and external validity while considering both individual and system level outcomes [41]. It serves to prioritize public health issues as it impacts real world settings, facilitating the translation of research into practice [42]. This framework has been applied in the evaluation of several health behaviour change interventions, specifically those that target physical activity, and diabetes [42-45]. The RE-AIM framework has been incorporated into the HEALD implementation and evaluation framework, through the use of a logic model and data matrix, that outlines evaluation questions, indicators and metrics, and data sources.

Our proposed evaluation activities that will inform dimensions of RE-AIM include systematic documentation of (a) usual care related to lifestyle counseling, (b) PCN organizational factors and strategies, (c) readiness and ability to implement HEALD through a checklist and interview guide administered to the Executive Directors, Chronic Disease Managers at each PCN, and (d) implementation facilitators and barriers identified by the Exercise Specialists. Data collected through the RE-AIM framework serves several evaluative functions: 1) determining the overall public health impact and translatability/applicability, 2) comparing the intervention’s public health impact across settings overtime, 3) comparing interventions across the various RE-AIM components, 4) assisting in the decision-making process of resource allocation for more effective programs, 5) providing information related to the level of implementation across the stages of research, and (6) providing valuable information to PCNs on the sustainability of long-term behaviour change (at patient and organization/system levels) beyond the intervention. All of these purposes have been highlighted in the diabetes literature as essential components to progress the research base in behaviour change [42,45-47].

##### Qualitative follow-up

To enhance to the validity of the quantitative findings and provide further information on the natural history (albeit retrospective) of the adoption of positive health behaviours for self-management, 10% of the participants (from each of the study groups at each of the PCNs) will be randomly selected to participate in a structured qualitative telephone interview at 6 months following the completion of the HEALD intervention. We hope to explicate their attitudes and behaviours related to the process of their healthy eating and active living behaviour change (or lack there of) and the program components.

### Analytic strategy

All statistical tests will be two-sided with a level of significance set at 0.05 to evaluate the effectiveness of the intervention. Baseline comparisons will be performed using univariate analysis of variance (ANOVA) for continuous variables and chi-square analyses for categorical variables. The main analysis of our study is to compare the physical activity of the HEALD-PCN intervention (On) group participants to the usual care (Off) group, from baseline to 6 months as our primary assessment time-point. Employing the intention-to-treat (ITT) approach, we will also assess differences in group changes from: (1) baseline to 3 months, (2) baseline to 6 months, (3) and 3 months to 6 months using generalized linear mixed-model analysis (GLMM). GLMM uses all available data and provide a valid analysis when data are missing at random. The models will use baseline values for each outcome variable as a covariate to reduce the residual standard error and account for regression to the mean. Potential confounding variables (i.e., age, sex and BMI) will be included as covariates in the models. All data will be analyzed using Statistical Package for the Social Sciences v.20, SPSS Inc., Chicago Illinois, USA and Stata SE 10.1, StatCorp, College Station, Texas, USA.

Based on the study’s design, we will account for 2 measurement points for the main dependent outcome (i.e., physical activity behavior at 6 months) across the study groups. In a 2 time-point, 2 group, repeated measures design with an a priori fixed and random effects model set to detect a moderate effect size (f = 0.25) while assuming a modest correlation (r = 0.5), the required sample size is 86 participants per group (power = 0.90; alpha = 0.05) [48-50]. Sample size was also estimated at 75 participants per group for average steps per day assuming an increase of 1,750 steps; that is, 8,250 versus 6,500 ± 3,300 steps/day for the intervention group over the control respectively (power = 0.90; alpha = 0.05). Our sample size estimate for our primary outcome is also sufficiently powered to detect a clinically meaningful difference between the groups of 0.5% for HbA1c assuming a baseline HbA1c of 7.2 ± 1.2%. This baseline estimation of HbA1c was derived from data provided by the Alberta Diabetes Surveillance System [17]. Given the length of follow-up period, we conservatively estimate that the rate of attrition during the intervention could reach 20% [51], and so our sample size was increased to 110 per group to account for the possibility of loss to follow-up.

## Discussion and conclusions

We have described the rationale and design of a trial that is pragmatic and applicable to examine the effectiveness and efficiency of a population-based strategy for two important components of chronic disease management among adults with T2DM. The HEALD-PCN intervention is a relevant approach to diabetes management, although some might argue that a self-management study for T2DM is not a novel intervention and that there is sufficient evidence for clinical improvements among this population after participating in an self-management interventions - we would agree. What is novel about this study is the implementation of the program in a “real world” setting, and by expanding the typical inter-disciplinary nature of the “diabetes team”; that is, participants will be recruited from primary care clinics in different areas of Alberta and Exercise Specialists who are now becoming more a part of the primary care disease management team, will deliver the program in routine care as it might be offered if not part of a research study. Moreover, we have established partnerships between a model for health care delivery (i.e., PCN) and a local resource (i.e., community recreation facility) that should support the achievement of some of the behavioural and clinical targets of diabetes management. This study will provide new information around clinical- and cost-effectiveness in the context of everyday primary care and thus it will significantly add to the current literature in health service delivery for diabetes self-management. The broader evaluation using the RE-AIM framework will produce timely and meaningful data for policy makers and health care providers interested in questions around the implementation and sustainability of this type of program. Having described the strengths of the current study, we also acknowledge the limitations of the HEALD-PCN study. First, although the design for the evaluation of HEALD-PCN is applicable from a health system delivery perspective (i.e., recruiting from patient registries to simulate regular patient invitations to PCN programs), it still remains a weaker design than a formal randomized controlled trial. Nevertheless, there is a need for pragmatic studies of this type, as questions related to the external validity should now take precedence over concerns of internal validity (i.e., the efficacy of the intervention has already been established) [18,52,53]. Second, as is often found in controlled quality improvement studies, the definition of usual care for the management of T2D is rather vague. For this study usual care may not reflect usual care in other primary care settings. To help answer any questions with respect to usual care in this setting, we will be collecting information from a system capacity perspective and from a patient perspective. As a result of the expected heterogeneity of usual care across the PCNs in this study, the generalizability of the findings will have to be contextualized to the individual PCNs if there are indeed substantial differences in what is deemed usual care. Nevertheless, the implementation of the study in four separate primary care settings allows for broader, comprehensive understanding with respect to issues of program effectiveness, applicability, feasibility and context. It is recognized that the currently available body of knowledge around this type of investigation handcuffs health policy makers and care providers when developing and initiating evidence-informed policies for new and existing health technologies and interventions [52-54].

In summary, healthy eating and active living remain cornerstones of T2DM management. Since the global prevalence of T2DM is expected to increase beyond previous estimates, best practices for promoting these self-management cornerstones at a population level are urgently needed. In addition to the T2DM population, the results of this study may provide valuable information relating to the implementation of other physical activity-based chronic disease prevention and management interventions.

## Abbreviations

ANOVA, Analysis of variance; HbA1c, Glycosylated haemoglobin; BMI, Body mass index; CDA, Canadian diabetes association; GI, Glycemic Index; GLMM, Generalized mixed-model analysis; GLTEQ, Godin leisure time exercise questionnaire; HEALD-PCN, Healthy eating and active living for diabetes in primary care networks; IPAQ, International physical activity questionnaire; MVPA, Moderate and vigorous; MET, Metabolic equivalents; PA, Physical activity; PCN, Primary care network; T2DM, Type 2 diabetes mellitus; RE-AIM, Reach, effectiveness, adoption, implementation and maintenance.

## Competing interests

None to declare.

## Authors’ contributions

The HEALD trial is part of a research program headed by JAJ. The lead investigators (STJ, JAJ and RCP) conceived the research questions and study design. STJ drafted the first version of the manuscript. CM, AS and LW participated in drafting this manuscript and will be responsible for supporting the implementation and evaluation of HEALD. All authors contributed to drafting this manuscript and approved the final version.

## Pre-publication history

The pre-publication history for this paper can be accessed here:

http://www.biomedcentral.com/1471-2458/12/455/prepub
